# Can Productive Aptitude and Age Affect Circulating Serotonin, Total Thyroid Hormones, and Cortisol Patterns in Cows?

**DOI:** 10.3390/vetsci11100471

**Published:** 2024-10-02

**Authors:** Giuseppe Bruschetta, Arianna Bionda, Renato Paolo Giunta, Giovanna Lucrezia Costa, Esterina Fazio, Patrizia Licata, Fabio Bruno

**Affiliations:** 1 Department of Veterinary Sciences, University of Messina, Viale Palatucci 13, 98168 Messina, Italy; giovannalucrezia.costa@unime.it (G.L.C.); esterina.fazio@unime.it (E.F.); patrizia.licata@unime.it (P.L.); fabio.bruno@unime.it (F.B.); 2 Department of Agricultural and Environmental Sciences, University of Milano, Via Celoria 2, 20133 Milan, Italy; arianna.bionda@unimi.it; 3 Experimental Zooprophylactic Institute of Sicily, Catania Area, Via Passo Gravina 195, 95125 Catania, Italy; renato.giunta@izssicilia.it

**Keywords:** serotonin (5-HT), thyroid hormones (THs), cortisol, cows, milk and meat aptitude, age, breed

## Abstract

**Simple Summary:**

In mammals, serotonin (5-hydroxytriptamine, 5-HT) is involved in many metabolic functions, including intestinal peristalsis, vasoconstriction, sleep, appetite, anxiety, and depression. The investigative hypothesis underlying this study was that different ages and productive aptitudes may affect 5-HT, thyroid hormones, and cortisol plasma concentrations and could explain possible interactions among them in cows. Our results showed significantly lower T3 concentrations in milk production aptitude cows than in meat production aptitude ones, a significant effect of age on T4 concentrations both in beef and dairy aptitude cows, and a positive and significant correlation for T4 with T3 and 5-HT in meat production-only cows. Monitoring of 5-HT, THs, and cortisol circulating concentrations could be a practical application to monitor the animal’s physiological adaptation processes.

**Abstract:**

Cattle productivity, whether in terms of meat yield or milk production, is intricately regulated by a multitude of factors. Among them, hormone concentrations play a significant role, reflecting the complex interplay between endocrine regulation and physiological processes that ultimately determine the efficiency and yield of production. High concentrations of 5-hydroxytriptamine (5-HT) are associated with a reduced metabolic load at the onset of lactation and a lower milk yield. Thyroid hormones (THs) and cortisol also affect several metabolic pathways, including carbohydrate, protein, and lipid metabolism. The aim of this study was to assess if milk or meat aptitudes and age influence circulating 5-HT, THs, and cortisol concentrations, investigating the possible interactions among these parameters. The research was performed on 46 healthy cows of three different breeds. Enzyme-linked immunosorbent assay (ELISA) methods were used to quantify circulating 5-HT and cortisol concentrations, and an immunochemiluminescent analyzer was used for THs. For parameters exhibiting non-normal distributions, an ANCOVA model using age, aptitude, and their interaction as fixed factors was applied. Significant lower T3 concentrations were recorded in dairy cows than in meat cows. Moreover, T4 significantly decreased with advancing age both in cows aimed at milk and meat production. Lastly, T4 was positively correlated with T3 and 5-HT in meat production-oriented cows.

## 1. Introduction

The use of biological indicators to assess animals’ physiological responses to adaptive stress as well as their recovery ability is the order of the day; this metabolic response can involve the secretion of serotonin or 5-hydroxytriptamine (5-HT) [1] and different hormones, such as cortisol [2,3] and thyroid hormones (THs) [4].

In mammals, 5-HT is synthetized from tryptophan through two enzymatic steps in the serotoninergic neurons as well as in some other tissues [5]. More than 98% of circulating 5-HT is located in platelets, which transport it into the peripheral blood [6]; together with lung and liver endothelial cells, platelets are also in charge of removing 5-HT from the blood [7]. 5-HT plays a role in many biological processes [8,9,10] such as glucose and lipid metabolism, calcium homeostasis, and the regulation of lactation in dairy cows [11,12,13,14]. During the non-lactating phase, the majority of 5-HT is synthesized and secreted by enterochromaffin cells within the intestine [15], while during lactation, ~50% of circulating 5-HT is produced and secreted from mammary epithelial cells [16]. It has been reported that lactating cows with high serum 5-HT concentrations show a reduced metabolic load at the onset of lactation, a lower milk yield, and reduced energy output via milk [12]. Recently, the existence of a coordinated serotonin–calcium feedback loop, involving endocrine and autocrine/paracrine mechanisms that regulate maternal and mammary calcium homeostasis, was recorded in dairy cows [11]. In addition, 5-HT has also been recognized as a marker of welfare and well-being in this species [1,7].

THs influence several metabolic pathways, including carbohydrate, protein, and lipid metabolism. This leads to increased basal energy expenditure [17,18], as supported by the positive correlation between circulating THs and energy balance observed in cattle [19]. TH concentrations vary according to physiological phases, with them being high in dry cows and significantly lower in the peripartum period and early lactation, when intense high milk production leads to substantial mobilization of metabolic reserves [20,21,22,23]. Specifically, in the first third of lactation, which is characterized by a negative energy balance, dairy cows show low T3 and T4 but an increase in fT3 concentrations [24]; additionally, it has been observed that T3 and T4 concentrations correlate negatively with milk yield [25].

The hypothalamic–pituitary–adrenal (HPA) axis and cortisol as its marker have crucial roles to play in the regulation of energy balance, food intake, and body weight, as recently recorded in growing beef calves [4]. Data obtained showed the interplay between THs and cortisol in energy metabolism suggesting that the thyroid and adrenocortical responses in finishing beef cattle serve as crucial endocrine signals for the initiation of energy utilization and the regulation of energy balance [26,27].

Today, specialized cattle breeds either accrete nutrients as meat (accretion type) or mainly transform nutrients into milk (secretion type) [28]. Data show age-related dynamics in changes to the level of dairy productivity in cows, revealing an average positive correlation between age and milk yield [29]. In light of this, we hypothesized that the differences in energy requirements across multiple metabolic directions, influenced by age and aptitude, could be associated with changes in 5-HT, THs, and cortisol concentrations in cattle.

Therefore, the scope of this study was to evaluate if beef or dairy aptitudes and age influence the circulating concentrations of these parameters, also investigating the possible interactions among them.

## 2. Materials and Methods

The research protocol was approved by the Ethical Committee of the Department of Veterinary Science, University of Messina, Italy (code n. 09/2024). The research complied with the guidelines of Good Clinical Practices (EMEA, 2000).

The present study included n. 46 adult healthy cattle (n. 14 Holstein dairy cows, n. 12 Brown Swiss dairy cows, and n. 20 Modicana x crossbred Limousine beef cows; the mean ± SD age was 42 ± 21 months), selected from a large group of 100 animals, reared on a commercial farm located in Catania, Italy (37°29′32″ N, 15°04′13″ E, 40 mt above sea level). Inclusion criteria for enrolled cows were: (i) the absence of reproductive diseases and (ii) the absence of any systemic or local inflammatory process and/or related antibiotic or anti-inflammatory pharmacologic treatment within the month before the start of sampling and throughout the entire experimental period.

At the time of sampling, the selected animals were assigned to one of the four age groups: 14–24 (n. 15 cows), 25–36 (n. 10 cows), 37–65 (n. 8 cows), or >65 months (n. 10 cows).

Dairy cows aged 14–24 months were primiparous at the 1st lactation, those of 25–36 months were multiparous at the 2nd or 3rd lactation, those of 37–65 months were multiparous at the 4th or 5th lactation, and those older than 65 months were multiparous at >5th lactation.

At the time of the study, all dairy cows were at the early lactation stage (45 ± 20 d) and not pregnant in anestrous post-partum. Each animal was milked twice daily at 4:30 and 16:30, with similar average milk production in both Holstein and Brown Swiss, equal to 28.5 ± 2 kg/head/day. The body condition score (BCS) for cows of the different breeds was: 2.5 ± 0.3 for the Holstein, 2.6 ± 0.3 for the Brown Swiss, and 2.9 ± 0.3 for the Modicana x crossbred Limousine breed.

All of the animals were bred under two traditional semi-extensive farming methods, according to their aptitude. Their indoor housing was a free-stall barn, with a space allocation of 4.5 m^2^/head and a straw-deep litter system. Dairy cows were fed with different diets composed of ad libitum polyphytic hay, sugar beet, and pasture supplementary feed for dairy cows (pellets) that was administered at 7:00 and 14:00 every day. Alternatively, pellets were replaced with farm-produced feed containing corn, soybeans, barley, bran, and vitamins. Beef cows were fed different diets composed of ad libitum polyphytic hay, sugar beet, and pasture; the concentrate (5 kg/head/meal) was administered at 7:00 and 14:00 every day. Pasture was available in the spring, for a minimum of 6 h during daylight, from 8:00 to 14:00; water was provided ad libitum.

### 2.1. Sample Collection

Blood samples were collected from the jugular vein at 8:00 at an environmental temperature of 18 °C (relative humidity: 0.3 ± 0.06) in April. An aliquot of blood was transferred to a 10 mL evacuated tube containing EDTA as an anticoagulant (K3-EDTA, Vacuette^®^, Greiner Bio-One, Kremsmünster, Austria), and used to evaluate plasma 5-HT; another aliquot was transferred to a 10 mL serum evacuated tube (Serum Clot Activator, Vacuette^®^, Greiner Bio-One, Kremsmünster, Austria), and used for serum THs and cortisol assays. The EDTA tubes were immediately refrigerated at 4 °C and subsequently (within 1–2 h) centrifuged at 4 °C for 10 min at 4500× *g* to obtain a platelet-poor plasma (PPP) fraction which is devoid of >96% of platelets [30,31]. After centrifugation, the PPP samples were harvested and stored at −80 °C until subsequent 5-HT analyses. The serum tubes were immediately refrigerated at 4 °C and centrifuged (within 1–2 h) for 10 min at 3000× *g*, and the supernatant serum was collected and stored at −20 °C until analyses of THs and cortisol within 2 weeks.

The TH system and TH-mediated signaling are known to play pivotal roles in the control of substrate utilization and are thus involved in thermoregulatory processes to maintain body temperature [4]. However, the environmental temperature of this experimental set was 18 °C and thus well within the thermoneutral comfort zone for the beef and dairy breed cows. Hence, the occurrence of the physiological processes that are usually triggered by environmental temperatures beyond either side of cows’ thermoneutral zone was excluded in the present study.

### 2.2. Analysis of Circulating 5-HT, THs, and Cortisol Concentrations

5-HT concentration was measured in duplicate in platelet-poor plasma (PPP) fraction using a commercial enzyme-linked immunosorbent assay (ELISA) kit (Ref. CEA808Ge CLOUD-CLONE CORP., Huston, TX, USA). 5-HT standards and plasma samples were handled according to the kit protocol. Briefly, 50 μL of each standard dilution, blank, and sample was added into the wells before adding 50 μL of Detection Reagent A. The plate was gently shaken and incubated for 1 h at 37 °C. Then, the solution was aspirated and washed with 350 μL of Wash Solution 3 times for 2 min. After the washing step, 100 μL of Detection Reagent B working solution was added to each well and the plate was incubated for 30 min at 37 °C. The aspiration/wash process was repeated 5 times before the addition of 90 μL of Substrate Solution. After incubation of 10 min at 37 °C, 50 μL of Stop Solution was added to each well and absorbance was read at 450 nm of wavelength (reference wavelength: 630 nm) using a microplate reader BIO-RAD 680 (BIO-RAD Laboratories, Segrate, Italy). Standard absorbance values were used to obtain the calibration curve from which the plasma 5-HT concentrations of the samples could be estimated. 5-HT concentration values were expressed in ng/mL. The sensitivity of the assay was 4.0 ng/mL. The average intra- and inter-assay coefficients of variation (CVs) were <10% and <12%, respectively.

The serum samples were evaluated in duplicate for total T3 and T4 hormones using an immunochemiluminescent analyzer (Immulite^®^ 2000, Siemens Medical Solutions, Diagnostics, Erlangen, Germany), according to the manufacturer’s instructions. All assays were validated for linearity using the cows’ serum before use. The intra-assay and inter-assay CVs were as follows: for T3, 12%, and 5.5% at T3 concentrations of 73 ng/dL and 171 ng/dL; for T4, 11.1% and 5.6% at T4 concentrations of 1.8 μg/dL and 16 μg/dL, respectively.

The serum samples were analyzed in duplicate for cortisol using a bovine competitive enzyme immunoassay (EIA) ELISA kit (Ref. LS-F10124 LSBio, Shirley, MA, USA). The assay’s sensitivity was 13.8 nmol/L, and the intra- and inter-assay CVs were 4% and 6.9%, respectively.

### 2.3. Statistical Analyses

JMP^®^ 17 software (Version 17., SAS Institute Inc., Cary, NC, USA, 1989–2023) was employed for statistical analyses. Descriptive statistics were generated for all of the variables and parameters exhibiting non-normal distributions that underwent logarithmic transformations. An ANCOVA model was then applied, with the blood concentration of hormones serving as the dependent variable and the animal’s aptitude (categorical variable), age (numerical variable expressed in months), and their interaction as fixed factors. The pairwise correlations among all of the analyzed blood parameters and age (in months) were calculated using Pearson’s linear correlation coefficient (r). Significance was attributed to *p*-values < 0.05.

## 3. Results

No significant differences among 5-HT, THs, and cortisol concentrations in the early lactating phases were observed between the two dairy breeds (Holstein and Brown Swiss), and therefore, all dairy animals were considered as a single group of n. 26 animals. The results of the ANCOVA models, including the *p*-values for the different fixed factors (in bold when significant) and the model’s R^2^, are shown in Table 1.

**Figure 1:**
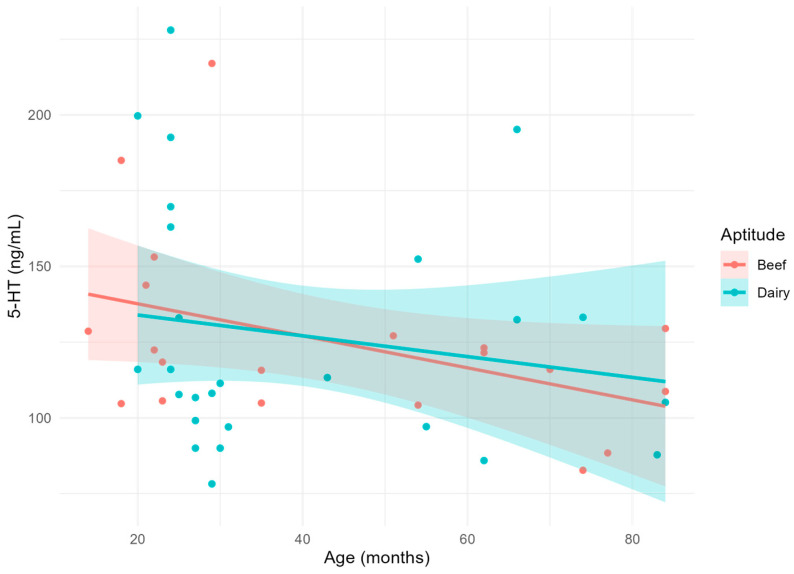
Scatterplot and linear relationship with confidence interval between age and serotonin or 5-hydroxytriptamine (5-HT).

Circulating 5-HT concentrations (Figure 1) were not significantly affected by age, aptitude, or their interaction. However, it was observed that 5-HT concentrations in younger animals (14–24 months) were higher for both aptitudes but more markedly in dairy animals.

**Figure 2:**
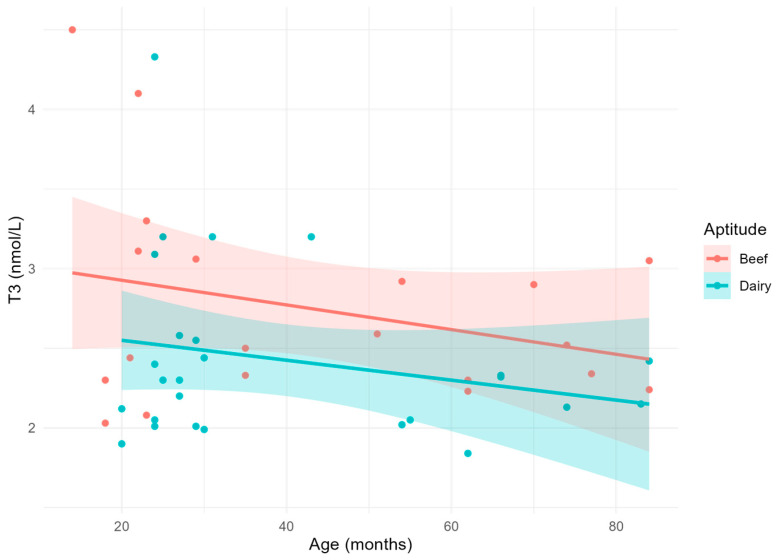
Scatterplot and linear relationship with confidence interval between age and T3.

Circulating T3 concentrations (Figure 2) were almost constant across the different ages, despite a tendency to slightly decrease with time in both groups. However, aptitude significantly affected T3 concentrations, with beef cows showing higher average values than the dairy ones (*p* = 0.0421).

**Figure 3:**
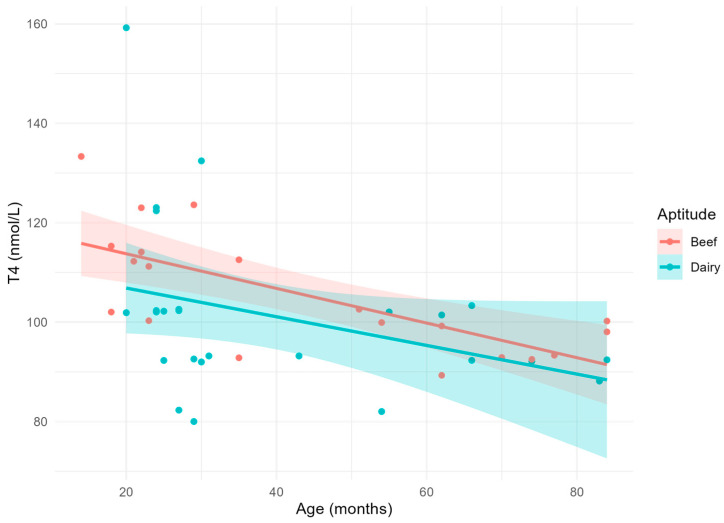
Scatterplot and linear relationship with confidence interval between age and T4.

Circulating T4 concentrations (Figure 3) significantly decreased with the increase in the cows’ age (*p* = 0.0008) without differences due to their aptitude.

**Figure 4:**
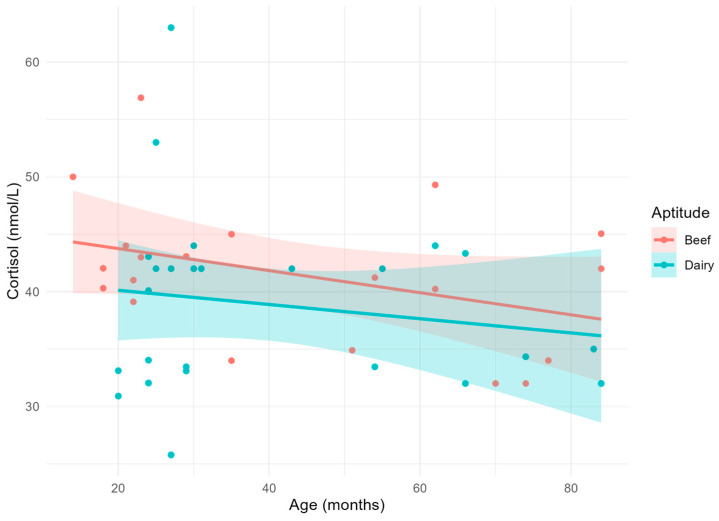
Scatterplot and linear relationship with confidence interval between age and cortisol.

Lastly, circulating cortisol concentrations (Figure 4) were not affected either by age, aptitude, or their interaction.

Correlations among the different parameters as well as age were estimated. No significant correlations were observed in dairy cows, whereas in beef cows, T4 was negatively correlated with age (r = −0.71, *p* = 0.0004) and positively correlated with T3 (r = 0.54, *p* = 0.0132) and 5-HT (r = 0.46, *p* = 0.0411).

## 4. Discussion

The most significant outcomes of this study were:

(1) Milk production aptitude (i.e., secretion type) was characterized by significantly lower T3 concentrations than meat production aptitude (i.e., accretion type) in cows;
(2) There is a significant effect of age on T4 concentration, which decreases with advancing age both in secretion and accretion types;
(3) A positive and significant correlation was recorded for T4 with T3 and 5-HT in the accretion type only.

The concentrations measured in the present study from both the dairy and beef cows are comparable to those previously reported for this species for circulating 5-HT [6,32], THs [17,33,34,35,36,37,38], and cortisol [4]. Therefore, the variations recorded may not indicate acute stress situations but rather punctual stimuli linked to the physiological systems of adaptation to beef/dairy production, among the other potential stressors. Moreover, the possible influence of circadian hormonal rhythm was limited by sampling all the cows enrolled in this study at the same time.

Differences in blood hormone concentrations, secretory patterns, and tissue sensitivity toward these hormones might be attributed to the different metabolic characteristics of individual breeds [28]. However, the previous literature does not provide a general description of the hormonal regulation of either accretion or secretion.

No significant effects of aptitude on PPP 5-HT concentrations were observed, but 5-HT levels were higher in younger animals compared to older ones for both types. This result is in accordance with our previous study on horses, in which higher PPP 5-HT concentrations were detected in foals compared to adult horses [39]. These variations are thought to be due to growth processes and greater 5-HT synthesis by enterochromaffin cells of the gastrointestinal tract in younger cows compared to adults, as hypothesized in horses [40].

Thyroid hormones play a key role in growing animals [4,41], with skeletal muscle proliferation, metabolism, differentiation, homeostasis, and growth being regulated by THs. In physiological conditions, TH stimulates both protein synthesis and degradation, and an alteration in TH concentration is often responsible for a specific myopathy [42]. What is more, T3 stimulates protein synthesis and plays a crucial role in cell metabolism, as it increases protein synthesis by stimulating gene transcription [43]. Therefore, when active protein synthesis takes priority over other metabolic alternatives, as in accretion-type (beef) cows, it is expected that significantly higher T3 concentrations will be found, as recorded in the present study.

The significant effect of age on T4 trend, which appeared to decrease with advancing age both in secretion and accretion types, could be due to alterations in TH economy along with changes in the responsiveness of various tissues to THs. The age-related resistance to THs can be attributed to decreased TH transport to tissues, decreased nuclear receptor occupancy, decreased activation of thyroxine to triiodothyronine, and alterations in TH-responsive gene expression [44].

The significantly lower T3 values observed in dairy cows than in beef ones confirmed the previously reported decrease in THs during the first third of lactation [45,46,47,48]. Indeed, this is the phase in which the cow experiences the maximum energy intake and reserves are mobilized for high milk production, so the decrease in TH concentration, especially T3, could favor the partitioning of nutrients between mammary and non-mammary tissue [49]. In addition, decreases in T3 concentration may incentivize efficient peripheral tissue metabolism during lactation [50] and the conservation of muscle mass [51]. However, it should also be noted that during lactation, water metabolism related to the mammary gland through the vascular system is physiologically increased, thus causing possible hemodilution of THs, as previously observed in lactating ewes for many hematological and biochemical substances [52]. Therefore, the reduction in serum T3 concentrations found in lactating dairy cows probably reflects the decreased hormone secretion rate due to energy deficiency, the large demand for these hormones by the mammary gland, or the physiological hemodilution occurring at the beginning of lactation [53].

With respect to the absence of significant differences in T3 and T4 concentrations recorded during the first stage of lactation between primiparous (group of 14–24 months) and multiparous (other groups) dairy cows, it is plausible that there was a large amount of individual variation in both the frequency of lactation and the total milk yield, which are known to be correlated with blood TH concentration [25,36,54,55], affecting the above individual variability. In addition, it is currently unknown whether TH concentration is influenced by milk yield or if THs may have regulatory functions as well. In contrast with our results, Fiore et al. [53] detected a decrease in T3 concentration at the beginning of lactation exclusively in cows in the second and third lactation, suggesting increased outer ring deiodination of T4 in the liver or mammary gland.

The significant positive correlations between T3 and T4 concentrations observed in beef cows are consistent with previous data observed in growing beef calves, reporting that changes in total iodothyronine concentration often follow the growth phase [4].

The significant positive correlation between 5-HT and T4 concentrations, observed only in the accretion type, is possibly due to the influence of the metabolic types, the characteristics of individual aptitudes, or different genetic merits. Circumstantial evidence suggests that THs can influence 5-HT synthesis [56]. Moreover, it is well known that reduced thyroid function (hypothyroidism) has been associated with decreased 5-HT production, whereas increased thyroid function (hyperthyroidism) can lead to increased 5-HT concentrations. It is therefore feasible that physiological changes, such as growth, influence cortisol concentrations in cows [57].

The constant and similar cortisol trend observed both in the secretion type (dairy cows) and accretion type (meat cows) supports the metabolic effect of corticosteroids on the mammary gland [58] and skeletal muscle [59]. Moreover, cortisol is a marker of stressful events when it is inappropriately high or low. The concentrations recorded during this study did not exceed normality ranges, indicating a condition of well-being, as previously recorded in periparturient [60] and postpartum dairy cows as well [61]. Moreover, cortisol has a generally negative effect on protein synthesis, inhibiting the construction of new proteins and promoting the degradation of existing proteins, especially in the context of muscle metabolism [59]. Therefore, physiological concentrations of cortisol recorded in this experimental protocol probably ensure the maintenance of skeletal muscle mass that is one of the main target tissues on which cortisol exerts its metabolic effects.

Finally, based on the results obtained in beef and dairy aptitude cows and the trend in the correlations, it is possible to hypothesize a different molecular mechanism in the role of thyroid hormones, cortisol, and 5-HT.

## 5. Conclusions

The measurement of 5-HT, THs, and cortisol plasma concentrations in secretion- and accretion-type cows provides information about the animal’s physiological adaptation processes. Furthermore, our results underline the importance of monitoring the hormonal status of beef and dairy aptitudes to understand when adjustments in regulatory mechanisms break through physiological limits, predisposing animals to metabolic problems throughout their productive life. However, further studies need to be carried out to better understand the effects of productive aptitude on circulating serotonin, total thyroid hormones, and cortisol in cows.

## Figures and Tables

**Table 1:** Results of the ANCOVA models, including the *p*-values for the different fixed factors (in bold when significant) and the model’s R^2^.

Parameter	Age (months)	Aptitude	Age × Aptitude	R^2^
5-HT (ng/mL)	0.0724	0.8434	0.6348	0.081
T_3_ (nmol/L)	0.1199	**0.0421**	0.9946	0.131
T_4_ (nmol/L)	**0.0008**	0.1095	0.6823	0.262
Cortisol (nmol/L)	0.1114	0.1324	0.6463	0.103

## Data Availability

The raw data supporting the conclusions of this article will be made available by the authors upon request.
